# Dimethyl 2,6-dimethyl-4-(2-nitro­phen­yl)pyridine-3,5-dicarboxyl­ate

**DOI:** 10.1107/S1600536811039626

**Published:** 2011-10-05

**Authors:** Juanjuan Zheng, Xueyuan Wang, Dongying Pang, Yan Sun, Wei Su

**Affiliations:** a Department of Chemistry, School of Science, Tianjin University, Tianjin 300072, People’s Republic of China; b TianJin Centralpharm Limited Company, Tianjin 300072, People’s Republic of China; c High Pressure Adsorption Laboratory, School of Chemical Engineering and Technology, Tianjin University, Tianjin 300072, People’s Republic of China

## Abstract

The title compound, C_17_H_16_N_2_O_6_, is a decomposition product of the hypertension drug nifedipine [systematic name: dimethyl 2,6-dimethyl-4-(2-nitro­phen­yl)-1,4-dihydro­pyridine-3,5-dicarboxyl­ate]. The dihedral angle between the nitro­sophenyl ring and the pyridine ring is 67.1 (5)°.

## Related literature

For the calcium antagonistic activity of compounds of the 1,4-dihydro­pyridine class, which inhibit the influx of Ca^2+^ ions through plasma membrane channels, see: Núnez-Vergara *et al.* (1994 ▶) and for their current use in the treatment of a variety of cardiovascular disorders such as angina and hypertension, see: Triggle *et al.* (1989 ▶); Hurwitz *et al.* (1991 ▶). For general background to derivatives of the dihydropyridine calcium channel blockers nifedipine [3,5-dimethyl 2,6-dimethyl-4-(2-nitrophenyl)-1,4-dihydropyridine-3,5-dicarboxylate] and nisoldpine [isobutyl methyl 2,6-dimethyl-4-(2-nitrophenyl)-1,4-dihydropyridine-3,5-dicarboxylate], see: Chen *et al.* (2010 ▶); Rowan & Holt (1996 ▶, 1997*a* ▶,*b* ▶); Schultheiss *et al.* (2010 ▶). For standard bond lengths, see: Allen *et al.* (1987 ▶). 
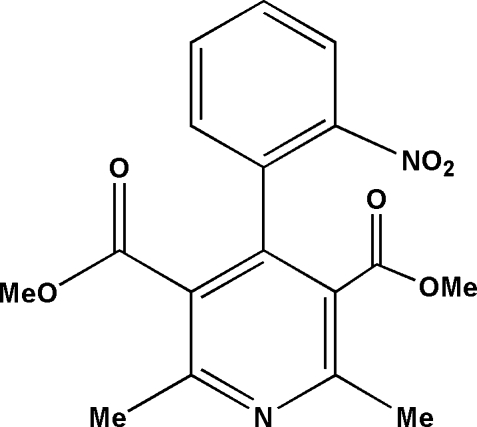


## Experimental

### 

#### Crystal data

C_17_H_16_N_2_O_6_
*M* *_r_* = 344.32
Triclinic, 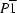

*a* = 7.578 (4) Å
*b* = 8.141 (4) Å
*c* = 14.235 (9) Å
α = 103.32 (2)°
β = 93.75 (5)°
γ = 105.39 (3)°
*V* = 816.4 (8) Å^3^
*Z* = 2
Mo *K*α radiation
μ = 0.11 mm^−1^
*T* = 298 K
0.20 × 0.18 × 0.12 mm

#### Data collection

Rigaku Saturn724 CCD diffractometer
Absorption correction: multi-scan (*CrystalClear*; Rigaku, 2005 ▶) *T*_min_ = 0.979, *T*_max_ = 0.987
8658 measured reflections
3843 independent reflections
2247 reflections with *I* > 2σ(*I*)
*R*_int_ = 0.047

#### Refinement

*R*[*F* ^2^ > 2σ(*F* ^2^)] = 0.038
*wR*(*F* ^2^) = 0.097
*S* = 1.03
3843 reflections
230 parameters
H-atom parameters constrained
Δρ_max_ = 0.21 e Å^−3^
Δρ_min_ = −0.29 e Å^−3^

### 

Data collection: *CrystalClear* (Rigaku, 2005 ▶); cell refinement: *CrystalClear*; data reduction: *CrystalClear*; program(s) used to solve structure: *SHELXS97* (Sheldrick, 2008 ▶); program(s) used to refine structure: *SHELXL97* (Sheldrick, 2008 ▶); molecular graphics: *SHELXTL* (Sheldrick, 2008 ▶); software used to prepare material for publication: *CrystalStructure* (Rigaku, 2005 ▶).

## Supplementary Material

Crystal structure: contains datablock(s) I, global. DOI: 10.1107/S1600536811039626/qm2031sup1.cif

Structure factors: contains datablock(s) I. DOI: 10.1107/S1600536811039626/qm2031Isup2.hkl

Supplementary material file. DOI: 10.1107/S1600536811039626/qm2031Isup3.cml

Additional supplementary materials:  crystallographic information; 3D view; checkCIF report

## Figures and Tables

**Fig. 1.**
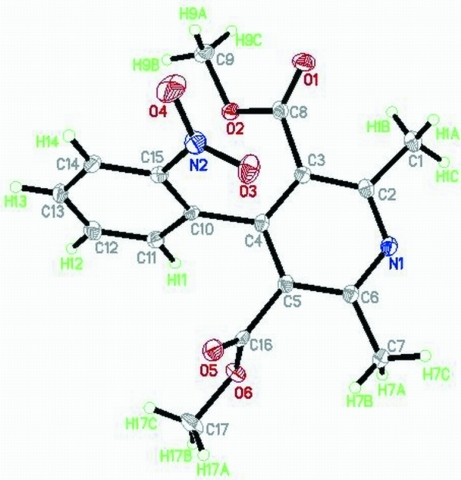
[3,5-dimethyl 2,6-dimethyl-4-(2-nitrophenyl)pyridine-3,5-dicarboxylate]

## supplementary crystallographic information

### Comment

Compounds of the 1,4-dihydropyridine class exhibit calcium antagonistic activity, as they inhibit the influx of Ca^2+^ ions through plasma membrane channels (Núnez-Vergara, Sunkel & Squella, 1994). Compounds of this class are currently being used in the treatment of a variety of cardiovascular disorders, such as angina and hypertension (Triggle *et al.*, 1989; Hurwitz *et al.*,  1991). Nifedipine [dimethyl 2,6-dimethyl-4-(2-nitrophenyl)-1,4-dihydropyridine-3,5-dicarboxylate], is the best known member of this class. The molecular structure of (I) is shown in Fig. 1. The bond lengths and angles are within normal ranges (Allen *et al.*, 1987). The dihedral angle between the nitrosophenyl ring and the pyridine ring is 67.1°.

### Experimental

The title compound was prepared by adding following steps. 1: Add 1 g nifedipine and 10 g (NH_4_)_2_S_2_O_8_ to the 100 ml acetone solution(50%). 2: Stir for 12 h at 30 °C.3:Regulate the solution to pH=8 with Na_2_CO_3_. The resulting solution was extracted with methylene chloride. The organic layer was dried over MgSO_4_ and evaporated under reduced pressure. Following washing the extract with water, crystals of suitable size for single-crystal analysis were recrystallized from methanol.

### Refinement

H atoms were positioned geometrically, with C—H = 0.93 and 0.96 A ° for aromatic and methyl H atoms, respectively, and constrained to ride on their parent atoms, with *U*_iso_(H) = xUeq(C), where *x* = 1.2 for aromatic and *x* = 1.5 for methyl H atoms.

### Crystal data

**Table:**

C_17_H_16_N_2_O_6_	*Z* = 2
*M**_r_* = 344.32	*F*(000) = 360
Triclinic, *P*1	*D*_x_ = 1.401 Mg m^−^^3^
*a* = 7.578 (4) Å	Mo *K*α radiation, λ = 0.71073 Å
*b* = 8.141 (4) Å	Cell parameters from 2919 reflections
*c* = 14.235 (9) Å	θ = 1.5–28.0°
α = 103.32 (2)°	µ = 0.11 mm^−^^1^
β = 93.75 (5)°	*T* = 298 K
γ = 105.39 (3)°	Prism, yellow
*V* = 816.4 (8) Å^3^	0.20 × 0.18 × 0.12 mm

### Data collection

**Table:**

Rigaku Saturn724 CCD diffractometer	3843 independent reflections
Radiation source: rotating anode	2247 reflections with *I* > 2σ(*I*)
multilayer	*R*_int_ = 0.047
Detector resolution: 14.22 pixels mm^-1^	θ_max_ = 27.9°, θ_min_ = 1.5°
ω and φ scans	*h* = −9→9
Absorption correction: multi-scan (*CrystalClear*; Rigaku, 2005)	*k* = −10→10
*T*_min_ = 0.979, *T*_max_ = 0.987	*l* = −18→17
8658 measured reflections	

### Refinement

**Table:**

Refinement on *F*^2^	Primary atom site location: structure-invariant direct methods
Least-squares matrix: full	Secondary atom site location: difference Fourier map
*R*[*F*^2^ > 2σ(*F*^2^)] = 0.038	Hydrogen site location: inferred from neighbouring sites
*wR*(*F*^2^) = 0.097	H-atom parameters constrained
*S* = 1.03	*w* = 1/[σ^2^(*F*_o_^2^) + (0.028*P*)^2^] where *P* = (*F*_o_^2^ + 2*F*_c_^2^)/3
3843 reflections	(Δ/σ)_max_ = 0.001
230 parameters	Δρ_max_ = 0.21 e Å^−^^3^
0 restraints	Δρ_min_ = −0.29 e Å^−^^3^

### Special details

**Table:**

Geometry. All e.s.d.'s (except the e.s.d. in the dihedral angle between two l.s. planes) are estimated using the full covariance matrix. The cell e.s.d.'s are taken into account individually in the estimation of e.s.d.'s in distances, angles and torsion angles; correlations between e.s.d.'s in cell parameters are only used when they are defined by crystal symmetry. An approximate (isotropic) treatment of cell e.s.d.'s is used for estimating e.s.d.'s involving l.s. planes.
Refinement. Refinement of *F*^2^ against ALL reflections. The weighted *R*-factor *wR* and goodness of fit *S* are based on *F*^2^, conventional *R*-factors *R* are based on *F*, with *F* set to zero for negative *F*^2^. The threshold expression of *F*^2^ > σ(*F*^2^) is used only for calculating *R*-factors(gt) *etc*. and is not relevant to the choice of reflections for refinement. *R*-factors based on *F*^2^ are statistically about twice as large as those based on *F*, and *R*- factors based on ALL data will be even larger.

### Fractional atomic coordinates and isotropic or equivalent isotropic displacement parameters (Å^2^)

**Table:**

	*x*	*y*	*z*	*U*_iso_*/*U*_eq_	
O1	0.76803 (15)	0.93198 (15)	0.92024 (8)	0.0333 (3)	
O2	0.84798 (13)	0.68996 (14)	0.85050 (7)	0.0240 (3)	
O3	0.40716 (15)	0.78829 (15)	0.70787 (8)	0.0344 (3)	
O4	0.63979 (19)	0.91761 (16)	0.64507 (10)	0.0520 (4)	
O5	0.13443 (14)	0.30250 (15)	0.61801 (7)	0.0279 (3)	
O6	0.18628 (14)	0.11367 (14)	0.70271 (7)	0.0259 (3)	
N1	0.26875 (17)	0.55143 (17)	0.95102 (9)	0.0218 (3)	
N2	0.54570 (19)	0.78817 (18)	0.66853 (9)	0.0283 (3)	
C1	0.5124 (2)	0.7905 (2)	1.05399 (10)	0.0256 (4)	
H1A	0.4799	0.8996	1.0549	0.038*	
H1B	0.6471	0.8165	1.0651	0.038*	
H1C	0.4582	0.7401	1.1054	0.038*	
C2	0.4386 (2)	0.6606 (2)	0.95644 (10)	0.0200 (3)	
C3	0.53855 (19)	0.65051 (19)	0.87679 (10)	0.0184 (3)	
C4	0.45649 (19)	0.52477 (19)	0.78913 (10)	0.0175 (3)	
C5	0.2806 (2)	0.4125 (2)	0.78490 (10)	0.0188 (3)	
C6	0.1892 (2)	0.4295 (2)	0.86757 (11)	0.0200 (3)	
C7	−0.0011 (2)	0.3140 (2)	0.86810 (11)	0.0278 (4)	
H7A	0.0077	0.2160	0.8954	0.042*	
H7B	−0.0653	0.2675	0.8013	0.042*	
H7C	−0.0701	0.3832	0.9080	0.042*	
C8	0.7278 (2)	0.7743 (2)	0.88633 (11)	0.0214 (3)	
C9	1.0316 (2)	0.8018 (2)	0.84997 (12)	0.0297 (4)	
H9A	1.0228	0.8948	0.8183	0.045*	
H9B	1.1038	0.7310	0.8141	0.045*	
H9C	1.0925	0.8554	0.9171	0.045*	
C10	0.55534 (18)	0.50220 (19)	0.70104 (10)	0.0176 (3)	
C11	0.6085 (2)	0.3487 (2)	0.67264 (10)	0.0226 (4)	
H11	0.5774	0.2612	0.7077	0.027*	
C12	0.7059 (2)	0.3213 (2)	0.59432 (11)	0.0267 (4)	
H12	0.7401	0.2154	0.5759	0.032*	
C13	0.7538 (2)	0.4482 (2)	0.54255 (11)	0.0263 (4)	
H13	0.8218	0.4296	0.4893	0.032*	
C14	0.7024 (2)	0.6014 (2)	0.56854 (11)	0.0237 (4)	
H14	0.7342	0.6887	0.5334	0.028*	
C15	0.60384 (19)	0.6254 (2)	0.64663 (10)	0.0202 (3)	
C16	0.19127 (19)	0.2747 (2)	0.69257 (11)	0.0200 (3)	
C17	0.1122 (2)	−0.0294 (2)	0.61593 (12)	0.0364 (4)	
H17A	−0.0139	−0.0318	0.5937	0.055*	
H17B	0.1105	−0.1415	0.6307	0.055*	
H17C	0.1898	−0.0116	0.5646	0.055*	

### Atomic displacement parameters (Å^2^)

**Table:**

	*U*^11^	*U*^22^	*U*^33^	*U*^12^	*U*^13^	*U*^23^
O1	0.0287 (7)	0.0182 (6)	0.0483 (8)	0.0042 (5)	0.0079 (5)	0.0014 (6)
O2	0.0200 (6)	0.0216 (6)	0.0293 (6)	0.0058 (5)	0.0056 (5)	0.0041 (5)
O3	0.0382 (7)	0.0388 (8)	0.0381 (7)	0.0221 (6)	0.0167 (6)	0.0170 (6)
O4	0.0680 (10)	0.0273 (8)	0.0727 (10)	0.0148 (7)	0.0355 (8)	0.0278 (8)
O5	0.0279 (6)	0.0343 (7)	0.0211 (6)	0.0059 (5)	0.0023 (5)	0.0102 (5)
O6	0.0320 (6)	0.0194 (6)	0.0223 (6)	0.0061 (5)	−0.0008 (5)	0.0004 (5)
N1	0.0245 (7)	0.0220 (7)	0.0214 (7)	0.0086 (6)	0.0074 (5)	0.0072 (6)
N2	0.0376 (9)	0.0251 (8)	0.0255 (8)	0.0098 (7)	0.0069 (6)	0.0111 (7)
C1	0.0318 (9)	0.0260 (9)	0.0212 (8)	0.0119 (8)	0.0040 (7)	0.0058 (7)
C2	0.0248 (8)	0.0195 (8)	0.0188 (8)	0.0109 (7)	0.0036 (6)	0.0057 (7)
C3	0.0200 (8)	0.0168 (8)	0.0211 (8)	0.0080 (7)	0.0040 (6)	0.0065 (7)
C4	0.0205 (8)	0.0173 (8)	0.0188 (8)	0.0094 (7)	0.0062 (6)	0.0072 (7)
C5	0.0210 (8)	0.0190 (8)	0.0188 (8)	0.0077 (7)	0.0052 (6)	0.0066 (7)
C6	0.0222 (8)	0.0190 (8)	0.0217 (8)	0.0082 (7)	0.0065 (6)	0.0072 (7)
C7	0.0247 (9)	0.0281 (10)	0.0287 (9)	0.0048 (8)	0.0119 (7)	0.0045 (8)
C8	0.0248 (8)	0.0223 (9)	0.0176 (8)	0.0074 (7)	0.0039 (6)	0.0051 (7)
C9	0.0192 (8)	0.0312 (10)	0.0363 (10)	0.0029 (7)	0.0059 (7)	0.0085 (8)
C10	0.0131 (7)	0.0195 (8)	0.0170 (8)	0.0008 (6)	0.0016 (6)	0.0034 (6)
C11	0.0219 (8)	0.0213 (9)	0.0241 (9)	0.0054 (7)	0.0041 (7)	0.0055 (7)
C12	0.0244 (9)	0.0258 (9)	0.0285 (9)	0.0101 (8)	0.0054 (7)	0.0005 (8)
C13	0.0201 (8)	0.0334 (10)	0.0210 (9)	0.0047 (8)	0.0067 (6)	0.0012 (8)
C14	0.0207 (8)	0.0276 (9)	0.0194 (8)	0.0001 (7)	0.0039 (6)	0.0071 (7)
C15	0.0185 (8)	0.0193 (8)	0.0207 (8)	0.0034 (7)	0.0023 (6)	0.0036 (7)
C16	0.0142 (7)	0.0234 (9)	0.0231 (9)	0.0041 (7)	0.0080 (6)	0.0073 (7)
C17	0.0424 (11)	0.0262 (10)	0.0303 (10)	0.0072 (9)	−0.0033 (8)	−0.0074 (8)

### Geometric parameters (Å, °)

**Table:**

O1—C8	1.2086 (18)	C5—C16	1.498 (2)
O2—C8	1.3392 (18)	C6—C7	1.500 (2)
O2—C9	1.4485 (18)	C7—H7A	0.9800
O3—N2	1.2218 (16)	C7—H7B	0.9800
O4—N2	1.2349 (16)	C7—H7C	0.9800
O5—C16	1.2107 (18)	C9—H9A	0.9800
O6—C16	1.3427 (19)	C9—H9B	0.9800
O6—C17	1.4481 (19)	C9—H9C	0.9800
N1—C2	1.342 (2)	C10—C15	1.393 (2)
N1—C6	1.344 (2)	C10—C11	1.394 (2)
N2—C15	1.478 (2)	C11—C12	1.385 (2)
C1—C2	1.506 (2)	C11—H11	0.9500
C1—H1A	0.9800	C12—C13	1.388 (2)
C1—H1B	0.9800	C12—H12	0.9500
C1—H1C	0.9800	C13—C14	1.381 (2)
C2—C3	1.403 (2)	C13—H13	0.9500
C3—C4	1.401 (2)	C14—C15	1.385 (2)
C3—C8	1.495 (2)	C14—H14	0.9500
C4—C5	1.390 (2)	C17—H17A	0.9800
C4—C10	1.502 (2)	C17—H17B	0.9800
C5—C6	1.402 (2)	C17—H17C	0.9800
			
C8—O2—C9	115.40 (13)	O2—C8—C3	111.74 (14)
C16—O6—C17	115.28 (12)	O2—C9—H9A	109.5
C2—N1—C6	120.11 (13)	O2—C9—H9B	109.5
O3—N2—O4	123.19 (15)	H9A—C9—H9B	109.5
O3—N2—C15	118.96 (13)	O2—C9—H9C	109.5
O4—N2—C15	117.85 (14)	H9A—C9—H9C	109.5
C2—C1—H1A	109.5	H9B—C9—H9C	109.5
C2—C1—H1B	109.5	C15—C10—C11	116.86 (13)
H1A—C1—H1B	109.5	C15—C10—C4	125.17 (14)
C2—C1—H1C	109.5	C11—C10—C4	117.94 (13)
H1A—C1—H1C	109.5	C12—C11—C10	121.28 (15)
H1B—C1—H1C	109.5	C12—C11—H11	119.4
N1—C2—C3	121.69 (15)	C10—C11—H11	119.4
N1—C2—C1	114.91 (13)	C11—C12—C13	120.22 (15)
C3—C2—C1	123.39 (14)	C11—C12—H12	119.9
C4—C3—C2	118.72 (14)	C13—C12—H12	119.9
C4—C3—C8	121.45 (13)	C14—C13—C12	119.93 (14)
C2—C3—C8	119.83 (14)	C14—C13—H13	120.0
C5—C4—C3	118.81 (13)	C12—C13—H13	120.0
C5—C4—C10	118.94 (14)	C13—C14—C15	118.90 (15)
C3—C4—C10	122.20 (13)	C13—C14—H14	120.5
C4—C5—C6	119.36 (14)	C15—C14—H14	120.5
C4—C5—C16	119.84 (13)	C14—C15—C10	122.80 (15)
C6—C5—C16	120.80 (14)	C14—C15—N2	116.56 (14)
N1—C6—C5	121.30 (14)	C10—C15—N2	120.61 (13)
N1—C6—C7	116.40 (13)	O5—C16—O6	123.94 (15)
C5—C6—C7	122.30 (14)	O5—C16—C5	125.59 (15)
C6—C7—H7A	109.5	O6—C16—C5	110.45 (13)
C6—C7—H7B	109.5	O6—C17—H17A	109.5
H7A—C7—H7B	109.5	O6—C17—H17B	109.5
C6—C7—H7C	109.5	H17A—C17—H17B	109.5
H7A—C7—H7C	109.5	O6—C17—H17C	109.5
H7B—C7—H7C	109.5	H17A—C17—H17C	109.5
O1—C8—O2	123.61 (15)	H17B—C17—H17C	109.5
O1—C8—C3	124.63 (14)		
			
C6—N1—C2—C3	−0.6 (2)	C5—C4—C10—C15	115.28 (17)
C6—N1—C2—C1	−179.75 (12)	C3—C4—C10—C15	−67.4 (2)
N1—C2—C3—C4	1.1 (2)	C5—C4—C10—C11	−66.86 (18)
C1—C2—C3—C4	−179.85 (13)	C3—C4—C10—C11	110.45 (17)
N1—C2—C3—C8	−179.70 (13)	C15—C10—C11—C12	0.4 (2)
C1—C2—C3—C8	−0.7 (2)	C4—C10—C11—C12	−177.67 (14)
C2—C3—C4—C5	−1.2 (2)	C10—C11—C12—C13	0.4 (2)
C8—C3—C4—C5	179.60 (13)	C11—C12—C13—C14	−0.7 (2)
C2—C3—C4—C10	−178.56 (13)	C12—C13—C14—C15	0.2 (2)
C8—C3—C4—C10	2.3 (2)	C13—C14—C15—C10	0.6 (2)
C3—C4—C5—C6	0.9 (2)	C13—C14—C15—N2	−177.47 (13)
C10—C4—C5—C6	178.33 (13)	C11—C10—C15—C14	−0.9 (2)
C3—C4—C5—C16	−178.88 (13)	C4—C10—C15—C14	176.99 (14)
C10—C4—C5—C16	−1.5 (2)	C11—C10—C15—N2	177.12 (13)
C2—N1—C6—C5	0.3 (2)	C4—C10—C15—N2	−5.0 (2)
C2—N1—C6—C7	−179.65 (13)	O3—N2—C15—C14	152.38 (14)
C4—C5—C6—N1	−0.5 (2)	O4—N2—C15—C14	−27.0 (2)
C16—C5—C6—N1	179.35 (13)	O3—N2—C15—C10	−25.7 (2)
C4—C5—C6—C7	179.50 (13)	O4—N2—C15—C10	154.89 (15)
C16—C5—C6—C7	−0.7 (2)	C17—O6—C16—O5	1.9 (2)
C9—O2—C8—O1	−3.1 (2)	C17—O6—C16—C5	−176.59 (11)
C9—O2—C8—C3	175.45 (12)	C4—C5—C16—O5	−70.6 (2)
C4—C3—C8—O1	131.12 (17)	C6—C5—C16—O5	109.63 (18)
C2—C3—C8—O1	−48.0 (2)	C4—C5—C16—O6	107.95 (15)
C4—C3—C8—O2	−47.44 (18)	C6—C5—C16—O6	−71.86 (17)
C2—C3—C8—O2	133.40 (14)		
